# Composition-Dependent Pseudocapacitive Behavior of MoO_3_–V_2_O_5_ Composite Thin Films in an Aprotic Electrolyte

**DOI:** 10.1021/acsomega.6c02074

**Published:** 2026-09-16

**Authors:** Nimbure Revansiddappa, Ramachandra Gokul, Boodu Anuragh, Muthu Selvakumar, MG Mahesha

**Affiliations:** Manipal Institute of Technology, 76793 Manipal Academy of Higher Education, Manipal 576104, India

## Abstract

Transition-metal oxide electrodes offer high theoretical capacitance for electrochemical energy storage. Yet, their practical application is fundamentally constrained by chemical instability in aqueous electrolytes, where high-valence metal centers undergo rapid hydrolytic dissolution. Here, we prepared MoO_3_–V_2_O_5_ composite thin films across different compositions. Structural characterization reveals a composition-dependent evolution from orthorhombic phases in the pure oxides to the coexistence of both the metal oxides in intermediate compositions. We employ an aprotic organic electrolyte (LiBF_4_ in propylene carbonate), which suppresses hydrolytic degradation pathways and enables stable operation over an expanded electrochemical window. Under these conditions, V_2_O_5_-rich thin-film electrodes exhibit pronounced pseudocapacitive behavior, delivering a specific capacitance of 25 F g^–1^ and capacitance retention of 89.7% at a current density of 1 A g^–1^ after 10,000 cycles along with a columbic efficiency of 98.4% in a symmetric device configuration. This work demonstrates a synergistic interaction between MoO_3_ and V_2_O_5_ phases, leading to enhanced charge storage behavior compared to the individual oxides.

## Graphical abstract

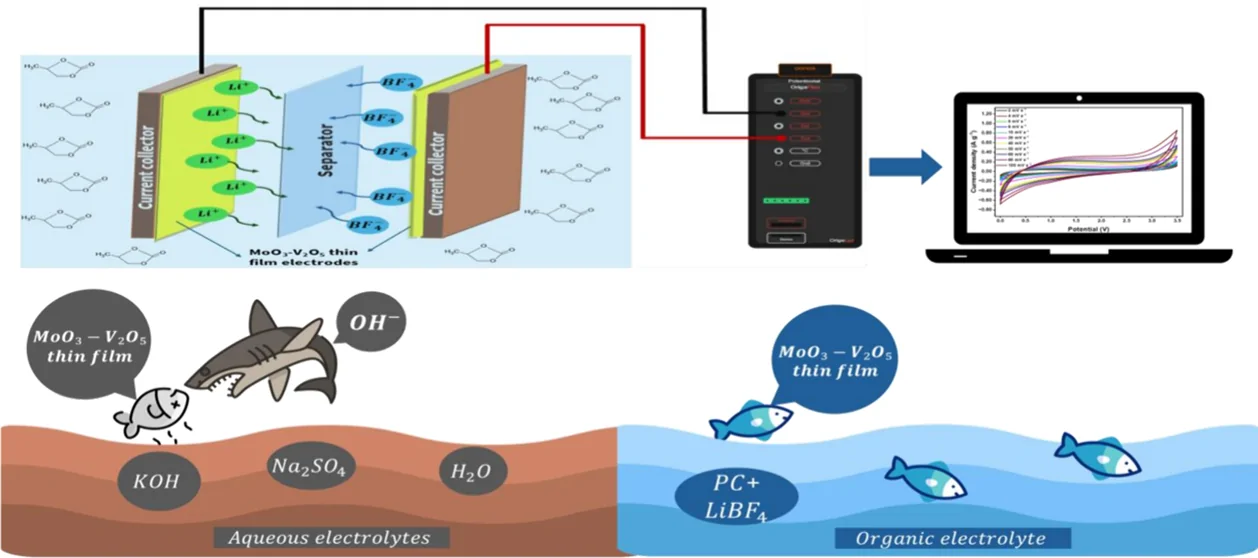

## 1 Introduction

The rapid expansion of renewable energy technologies such as solar and wind has increased the need for efficient energy storage systems.^1^ However, the intermittent nature of these resources necessitates efficient energy storage systems capable of storing excess energy and delivering it on demand. Electrochemical energy storage technologies including lithium-ion batteries,^2,3^ sodium-ion batteries,^4,5^ fuel cells,^6^ and supercapacitors^7^ have emerged as key solutions owing to their high energy conversion efficiency, scalability, and compatibility with renewable energy systems. Among these energy storage solutions electrochemical supercapacitors (SCs) emerged as essential devices due to their high power density, fast charge–discharge capability, and long cycle stability, which makes them better than conventional batteries in applications that require instantaneous energy delivery.^8^ Unlike batteries, which depend upon slow bulk ion diffusion, SCs store charge through a combination of electric double layer formation and fast reversible surface redox reactions, enabling exceptional rate performance and durability.^1^ Figure 1 illustrates the three principal charge storage mechanisms in supercapacitors, namely, electric double layer capacitance (EDLC)^9^ arising from electrostatic ion accumulation, pseudo capacitance^10^ originating from fast surface or near surface redox reactions, and hybrid supercapacitors^11^ that integrate both the processes. As global energy consumption rises and sustainable power becomes increasingly essential, developing advanced electrode materials for high-performance SCs remains a critical scientific and technological priority. Among the various electrode material classes explored for SCs include carbon-based materials,^12^ conducting polymers,^3,5,13^ and transition metal oxides (TMOs).^4,14^ The performance of the device is intimately governed by the choice of electrode and its electrochemical properties. TMOs represent a diverse and fascinating class of pseudocapacitive materials with versatile electronic structures arising from partially filled d-orbitals.^15^ The metal oxygen bonding in these compounds can vary from nearly ionic to highly covalent, enabling multiple oxidation states and reversible redox reactions that are essential for charge storage.^16^ Among TMOs, RuO_2_ is thought to be an optimal pseudocapacitive electrode material owing to its high theoretical capacitance and rapid faradaic redox reactions; however, the high cost and toxicity to the environment seriously hinder its practical application in SCs.^17^ This has driven the research community towards earth-abundant, cost-effective TMOs such as MnO_2_, NiO, Co_3_O_4_, MoO_3_, and V_2_O_5_ as viable alternatives. Among these, molybdenum trioxide (MoO_3_) and vanadium pentoxide (V_2_O_5_) have attracted considerable attention due to their layered structures, multiple oxidation states, and relatively low cost.^18^ MoO_3_ has been explored as pseudocapacitive electrode material for improvement in energy density owing to its unique layered structure composed of MoO_6_ octahedra with van der Waals interactions, which helps in insertion and extraction of ions.^19^ Molybdenum possesses multiple accessible oxidation states ranging from +2 to +6, which facilitates complex, multi electron faradaic charge transfer mechanisms.^20^ MoO_3_ exists in three primary polymorphs: orthorhombic (α), monoclinic (β), and hexagonal (h). The most thermodynamically stable and commonly utilized form is α-MoO_3_. Its structure supports reversible Mo^6+^/Mo^4+^ redox transitions, enabling efficient pseudocapacitive charge storage.^21^ As a result, MoO_3_ shows high theoretical specific capacity exceeding 1117 mAh g^–1^.^22^ However, translating this immense theoretical potential into practical performance is hindered by inherent material limitations. Pristine MoO_3_ suffers from sluggish faradaic redox kinetics, low electrical conductivity, and lack of structural integrity during cycling at high rates.^23^ V_2_O_5_ is another pseudocapacitive TMO which offers multivalency, different oxidation states from V^2+^ to V^5+^, which promotes high-capacity ion intercalation and rich pseudocapacitive behavior.^24^ The well-defined layered crystal structure accommodates a variety cations including H^+^, L^+^, Na^+^, and K^+^ through intercalation-type pseudocapacitance making it attractive for asymmetric and hybrid SCs application.^25,26^ V_2_O_5_ is limited by low power density and minimal cyclic stability because of low ionic diffusion rate.^27^

**Figure 1:**
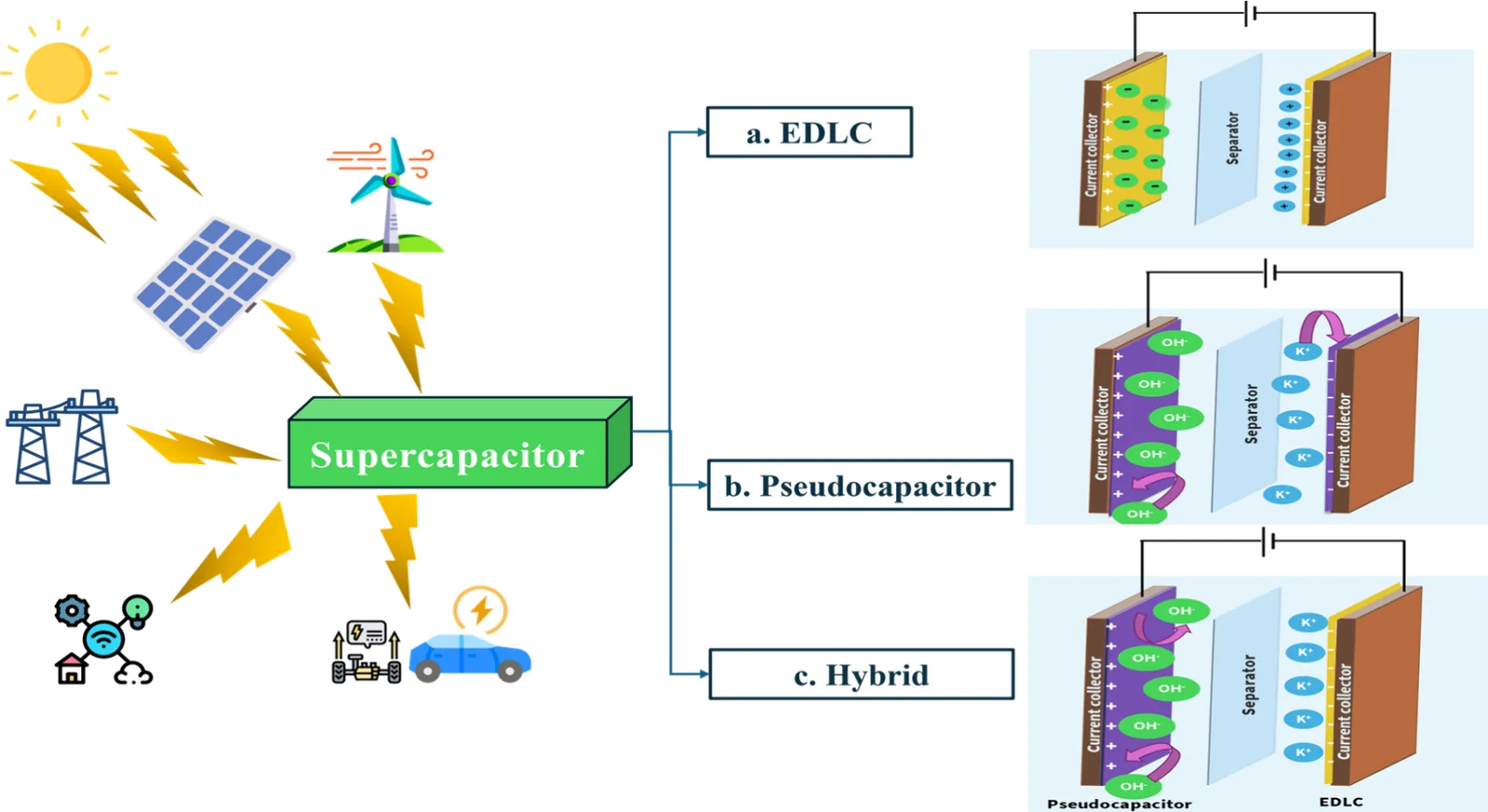
Supercapacitors in renewable energy storage and their classification into a. EDLC, b. pseudocapacitor, and c. hybrid types.

While pristine V_2_O_5_ and MoO_3_ have been extensively researched, MoO_3_–V_2_O_5_ composite thin films remain largely unexplored for energy-storage applications. The existing literature has shown that MoO_3_–V_2_O_5_ composite thin films exhibit better performance for gas sensing, such as NO_2_ detection.^28^ Despite these promising attributes, most studies of MoO_3_ and V_2_O_5_ are limited to powder or nanostructured systems in aqueous electrolytes. Each of these oxides individually offers complementary electrochemical merits such as MoO_3_ contributing a unique layered structure with favorable Mo redox transitions, while V_2_O_5_ provides a wide accessible potential window through V^5+^/V^4+^/V^3+^ redox activity. A composite of these two materials is therefore expected to yield synergistic enhancements in charge storage through the simultaneous utilization of both Mo and V redox couples, potentially broadening the operational potential window and improving the overall pseudocapacitive performance.^29^ The present work investigates MoO_3_–V_2_O_5_ composite thin films as electrode materials for supercapacitors.

Our preliminary research revealed that MoO_3_–V_2_O_5_ composite thin films were catastrophically unstable in aqueous environments. Upon exposure to a standard aqueous electrolyte (1 M Na_2_SO_4_ or 1 M KOH), we observed an immediate and macroscopic dissolution of the active material, making standard electrochemical characterization impossible. This rapid degradation is attributed to the intrinsic amphoteric nature of high-valence Mo^6+^ and V^5+^ centers, transforming the solid oxide network into highly soluble anions of molybdate (MoO_4_^2–^) and vanadate (VO_3_^–^) that easily diffuse into the surrounding medium.^30^ Consequently, to overcome this fundamental hydrolytic instability and access the intrinsic pseudocapacitive properties of the prepared thin-film electrodes, we transitioned to an anhydrous organic electrolyte system (1 M LiBF_4_ in propylene carbonate), which not only preserves structural integrity but also expands the operating voltage window, thereby enhancing energy density.^31^ In the present study, the MoO_3_–V_2_O_5_ thin films were deposited by spray pyrolysis, and their electrochemical properties were systematically evaluated in LiBF_4_/propylene carbonate organic electrolyte, with the aim of establishing the fundamental electrochemical behavior of this composite system and demonstrating its viability as a next-generation thin-film supercapacitor electrode.

## 2 Experimental Methods

### 2.1 Preparation of Thin Films

MoO_3_–V_2_O_5_ composite thin films were synthesized using the spray pyrolysis technique, employing computer interfaced HOLMARC spray pyrolysis equipment (Model No. HO-TH-04).^32^ High purity precursors ammonium heptamolybdate ((NH_4_)_6_ Mo_7_ O_24_·4H_2_ O, AHM, ≥ 99% purity, Merck) and ammonium metavanadate (NH_4_ VO_3_, AMV, ≥ 99% purity, Merck) were dissolved in deionized water to prepare 0.01 M AHM and 0.07 M AMV solutions. Although the molarities are different, both solutions give an identical metal atom concentration of 0.07 M (AHM contains 7 Mo atoms per molecule; AMV contains 1 V atom per molecule), which provides an equal metal atom equivalence approach for precise compositional tailoring.

The resulting precursor solution was atomized using compressed air as the carrier gas with a pressure of 1 bar and delivered at a constant flow rate of 1 mL min^–1^. The nozzle to substrate distance was fixed at 17 cm to ensure uniform droplet distribution and controlled film growth. The precursor solution was sprayed onto cleaned fluorine-doped tin oxide (FTO) substrates maintained at 425 °C; upon reaching the heated substrate, the aerosol droplets underwent rapid solvent evaporation followed by thermal decomposition of the precursor salt, leading to the formation of oxide films. The decomposition results in nucleation of MoO_3_ and V_2_O_5_ phases followed by their growth into a continuous thin film. Films with different Mo:V ratios were grown, and these samples were named as shown in Table 1. All the deposition parameters were kept constant for different compositions. The film preparation procedure is depicted in Figure 2. The decomposition reactions of ammonium heptamolybdate and ammonium metavanadate are given below in eqs 1 and ([Disp-formula eq2]).

1 $${\left({\mathrm{NH}}_{4}\right)}_{6}{\mathrm{Mo}}_{7}O_{24}\cdot 4H_{2}O\to 7{\mathrm{MoO}}_{3}+6{\mathrm{NH}}_{3}↑+7H_{2}O↑$$

2 $$2{\mathrm{NH}}_{4}{\mathrm{VO}}_{3}\to V_{2}O_{5}+2{\mathrm{NH}}_{3}↑+H_{2}O↑$$

**Figure 2:**
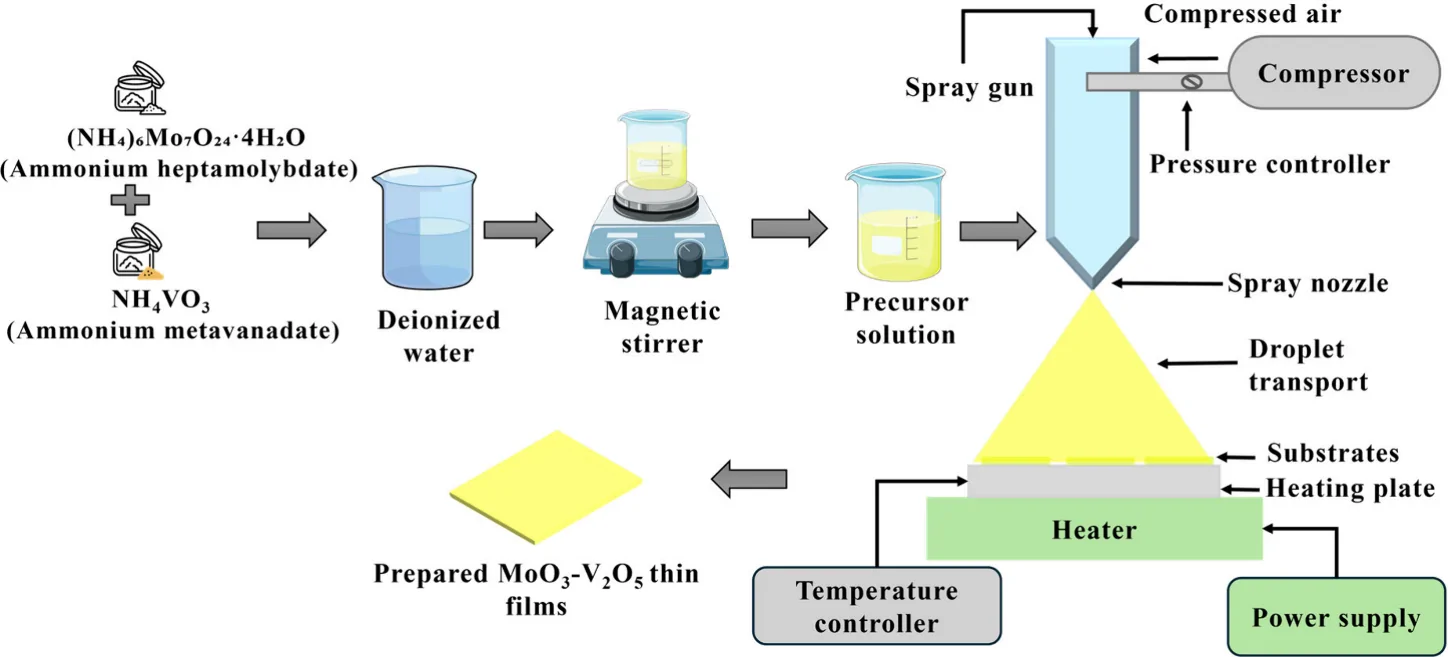
Schematic diagram of MoO_3_–V_2_O_5_ thin film deposition by Spray pyrolysis.

**Table 1:** Compositional Ratio of Mo:V

Sample name	Nominal Mo:V	From EDS $\frac{\mathrm{Mo}}{\left(\mathrm{Mo}+V\right)}$ (%)
M100	100:0	100
M80	80:20	78.78
M60	60:40	58.36
M50	50:50	48.90
M40	40:60	39.12
M20	20:80	19.57
V100	0:100	0

### 2.2 Material Characterizations

Structural, morphological, electrical, and electrochemical properties of all the prepared thin film samples were comprehensively analyzed using diverse characterization techniques. High resolution X-ray diffraction (HRXRD) measurements were conducted using a Rigaku Smart Lab diffractometer equipped with a 9 kW rotating anode Cu–Kα source (λ = 1.5406 Å) and operated at a step rate of 2° min^–1^. Surface morphology and cross-sectional thickness were examined using a Thermo Scientific apreo 2S FESEM. Raman measurements were performed using a LabRAM Raman spectrometer equipped with a 532 nm excitation laser. Electrical measurements were performed using a linear four-probe configuration connected to a Keithley 2450 source meter, operated at a constant current of 1 mA with a 20 V compliance limit to determine sheet resistance and resistivity. All the electrochemical characterizations including cyclic voltammetry, galvanostatic charge–discharge, and electrochemical impedance spectroscopy were carried out using an OrigaFlex-OGF05A potentiostat, enabling detailed evaluation of the pseudocapacitive response and charge storage performance of the thin film electrodes.

## 3 Results and Discussion

### 3.1 X-ray Diffraction (XRD)

X-ray diffraction (XRD) was used to analyze the phase formation, crystal structure of MoO_3_–V_2_O_5_ thin films with systematically varied Mo:V metal atomic ratio. Figure 3(a) presents the XRD pattern for all compositions in the 2θ range of 10°-70°. The M100 sample displays diffraction peaks associated with orthorhombic α-MoO_3_ (JCPDS 01-0706), including reflections at 12.8°, 23.3°, 25.7°, 27.3°, 38.9°, 49.2°, and 55.5°, indexed to the (020), (110), (040), (021), (060), (230), and (081) planes, respectively.^33^ The V100 sample exhibits characteristic reflections of orthorhombic α-V_2_O_5_ (JCPDS 01-070-8747), with prominent peaks located at 20.3°, 26.1°, 31.0°, 34.3°, 41.2°, 47.4°, and 51.2°, corresponding to the (001), (110), (040), (131), (002), (012), and (051) planes, respectively.^34^ The intermediate films (M20-M80) show diffraction peaks of both MoO_3_ and V_2_O_5_, which confirms the coexistence of two oxides. The intended compositional tailoring is directly demonstrated by the gradual variation in peak intensities, where MoO_3_ peaks become prominent in MoO_3_ rich samples (M60, M80, M100) and V_2_O_5_ peaks dominate in V_2_O_5_ rich samples (V100, M20, M40), and a similar trend was also reported by Mane A et al.^28^ The intensity of the V_2_O_5_ (001) peak at 20.3° is much higher than that of the corresponding MoO_3_ peaks. This intensity difference results not only from the differences in crystallinity but also from the intrinsically higher X-ray scattering factor and strong structure factor of α-V_2_O_5_.^28^ A closer view of the most intense peak of V_2_O_5_ shown in Figure 3(b) reveals a clear and systematic peak shift across the compositional series. The characteristic V_2_O_5_ peak at 20.16° in the V100 film progressively shifts toward higher diffraction angles as MoO_3_ content increases, indicating a gradual contraction of the lattice. The observed peak shifts may arise from strain-mediated properties between the two oxide phases. Crystallite size (D), dislocation density (δ), and microstrain (ε) were quantified to evaluate the microstructural changes across the series, and these parameters were extracted from the most intense diffraction peak of each sample. The crystallite size was estimated using the Scherrer formula **(3)**, where *K* is the shape factor, *λ* is the wavelength of Cu–K_α_ radiation, *β* is the full width at half maximum (FWHM), and *θ* is the diffraction angle.^35^ The dislocation density was calculated using relation **(4)**, which provides an approximate measure of the defect concentration within the crystallites.^36^ Microstrain (ε) was determined from the peak broadening according to relation **(5)**.^37^ All the calculated structural parameters are summarized in Table 2.

3 $$D=\frac{K\lambda }{\beta \cos\theta }$$

4 $$\delta =\frac{1}{D^{2}}$$

5 $$\varepsilon =\frac{\beta }{4\tan\theta }$$

**Figure 3:**
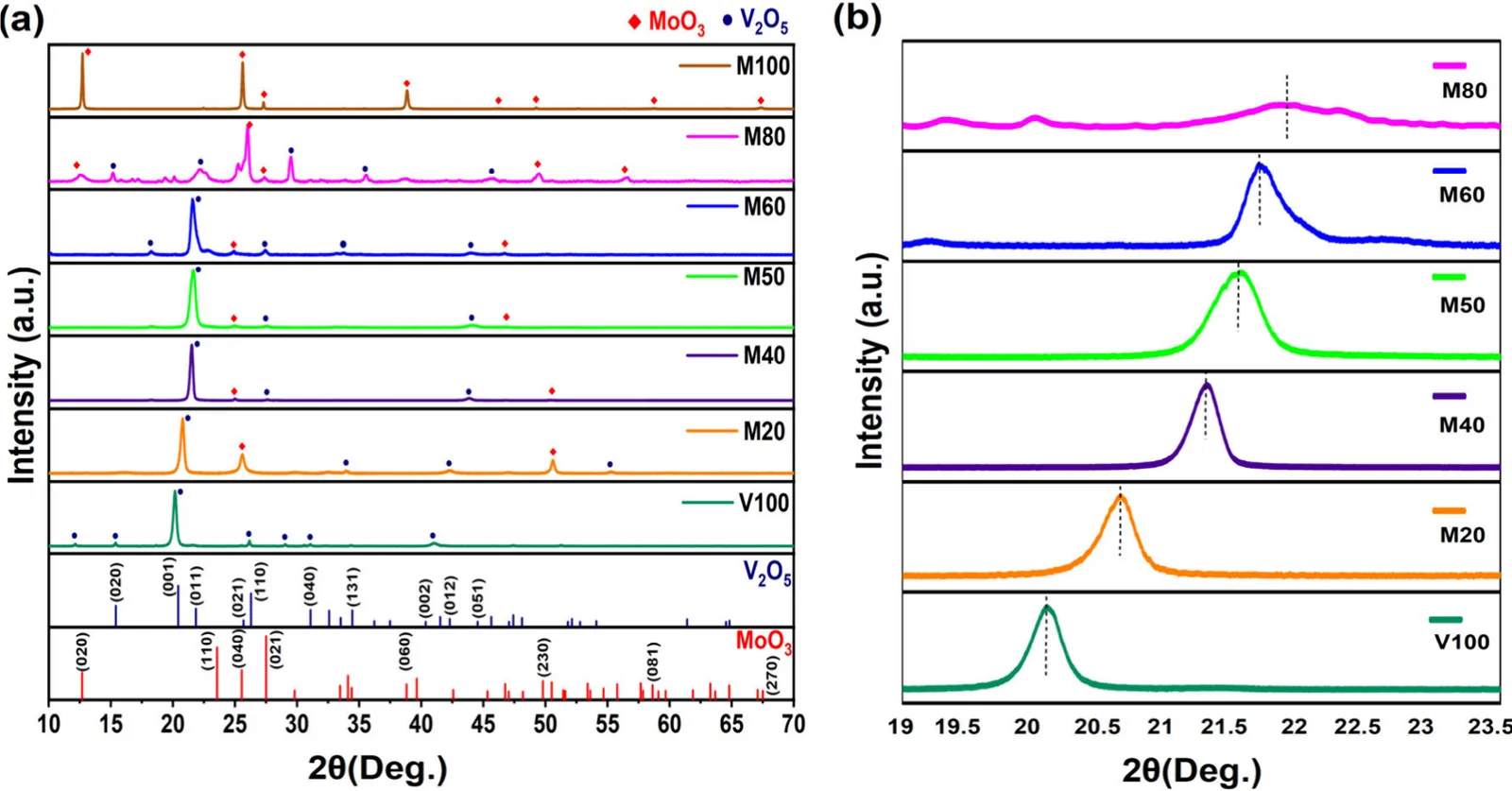
(a) XRD patterns of prepared MoO_3_–V_2_O_5_ thin films across different compositions, (b) Magnified image showing peak shift.

**Table 2:** Films Crystallite Size, Dislocation Density, and Microstrain

Sample	hkl	2θ (Deg.)	β (Deg.)	D (nm)	δ (× 10^–3^ nm^–2^)	ε (× 10^–3^)
M100	(040)	25.6	0.17	50.22	0.39	3.25
M80	(021)	25.9	0.41	20.63	2.36	7.81
M60	(011)	21.6	0.33	25.41	2.78	7.61
M50	(011)	21.6	0.47	17.83	5.97	10.82
M40	(011)	21.5	0.29	29.12	1.30	6.66
M20	(001)	20.8	0.37	22.93	2.34	8.76
V100	(001)	20.2	0.34	24.92	0.64	8.30

### 3.2 X-ray Photoelectron Spectroscopy Analysis

X-ray photoelectron spectroscopy (XPS) was used to identify the chemical state of elements in the prepared thin films. Figure 4(a) shows the survey spectrum of M20 composition thin film. The carbon C 1s peak at 284.6 eV was used to calibrate the XPS spectra. The different peaks in the survey spectrum were identified, and they confirmed the presence of Mo, V, and O elements. The core spectrum Mo 3d is shown in Figure 4(b) and exhibits a well-defined doublet at 232.56 eV (Mo 3d_5/2_) and 235.69 eV (Mo 3d_3/2_) which agrees well with the literature.^38,39^ The doublet peaks at the Mo^6+^ show a peak separation of 3.13 eV^40^ which confirms the presence of MoO_3_ in the film. The V 2p core spectrum shown in Figure 4(c) displays dominant peaks at 517.2 eV (V 2p_3/2_) and 524.5 eV (V 2p_1/2_), corresponding primarily to the V^5+^ state, which agrees well with the literature.^41,42^ The doublet peaks at V^+5^ show a peak separation of 7.2 eV, which confirms the presence of V_2_O_5_ in the film.^43^ The O 1s core spectrum can be deconvoluted into two components as shown in Figure 4(d), a main peak at 530.5 eV associated with lattice oxygen (Mo–O–Mo, V–O–V, and Mo–O–V bonds), and a higher binding energy component at 531.6 eV attributed to the hydroxyl group. The detailed peak positions, full width at half maximum (FWHM), relative peak areas, and corresponding chemical assignments obtained from the deconvoluted Mo 3d, V 2p, and O 1s spectra are summarized in Table 3. The composition of the M20 sample was further confirmed by the data obtained from the XPS spectra; using eq 6, the atomic fraction of the elements present in the sample was calculated, where A_*x*_ is the area of the peak of the element *x*, and RSF_*x*_ is the relative sensitive factor of the element.^44^ The atomic% calculated is 4.50, 6.60, and 88.9% for Mo, V, and O, respectively.

6 $$Atomic,fraction,of,x=\frac{\frac{A_{x}}{RSF_{x}}}{\underset{i}{\sum }\frac{A_{i}}{RSF_{i}}}$$

**Table 3:** XPS Peak Fitting Parameters for Mo 3d, V 2p, and O 1s Core Levels of the M20

Core Level	Peak position (eV)	FWHM (eV)	Area	Assignment
Mo 3d_5/2_	232.56	1.43	45356.16	Mo^6+^ (MoO_3_)
Mo 3d_3/2_	235.69	1.47	30409.25	Mo^6+^ (MoO_3_)
V 2p_3/2_	517.2	1.63	78214.31	V^5+^ (V_2_O_5_)
V 2p_1/2_	524.4	2.62	34763.2	V^5+^ (V_2_O_5_)
O 1s	530.5	1.62	187724.7	Lattice oxygen
	531.6	2.22	274293.4	Hydroxyl/Vacancy

**Figure 4:**
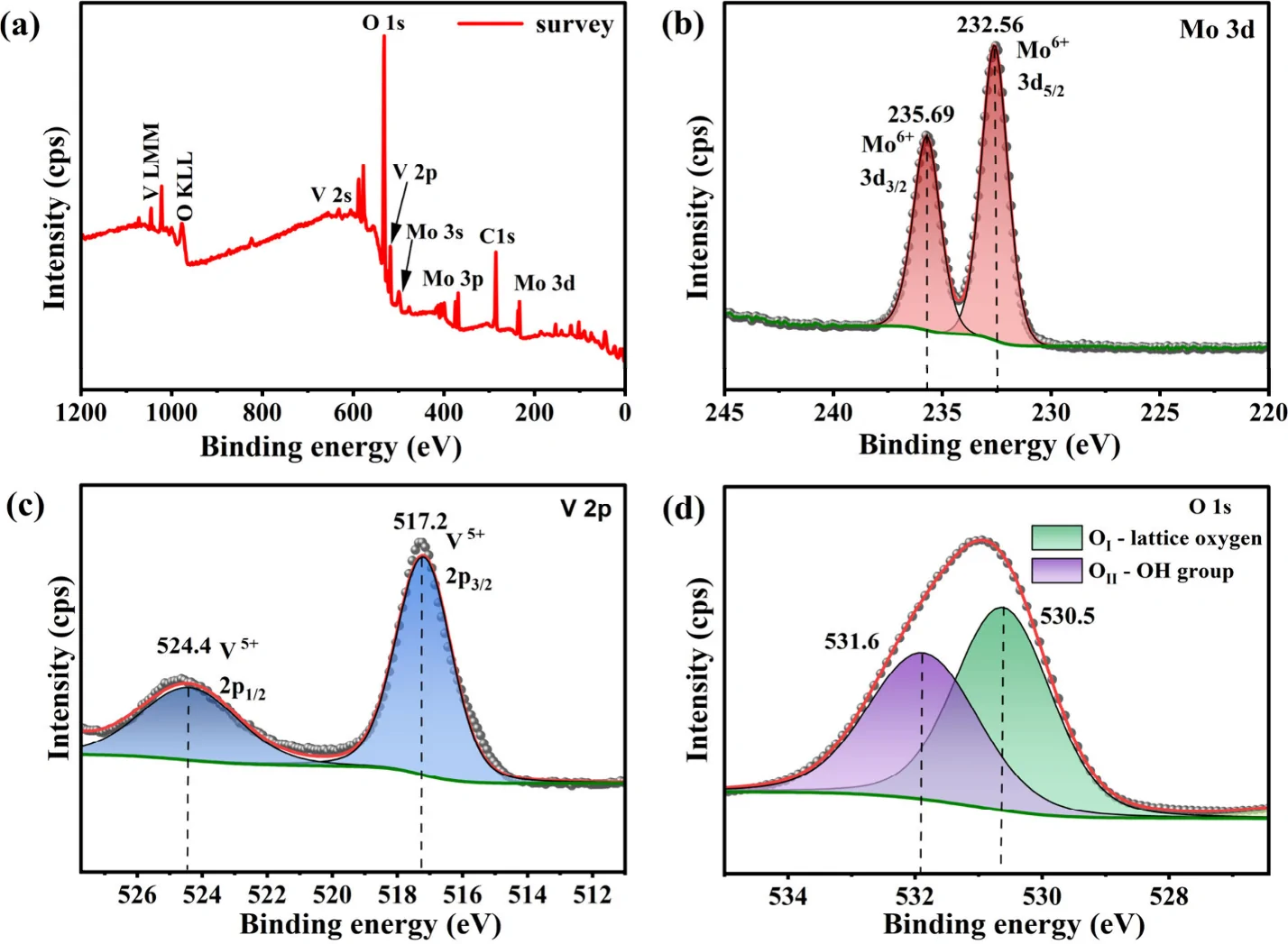
XPS spectra of M20 sample. (a) Survey spectrum, core spectra of (b) Mo, (c) V, (d) O 1s energy levels.

### 3.3 Raman Analysis

Raman spectroscopy was also employed to characterize the structural evolution of MoO_3_–V_2_O_5_ thin films across all the compositions. The Raman spectrum shown in Figure 5 displayed prominent characteristic peaks at 117 cm^–1^, 129 cm^–1^, 158 cm^–1^, 197 cm^–1^, 218 cm^–1^, 243 cm^–1^, 285 cm^–1^, 292 cm^–1^, 337 cm^–1^, 378 cm^–1^, 471 cm^–1^, 667 cm^–1^, 820 cm^–1^, and 996 cm^–1^, which corresponds to orthorhombic α-MoO_3_ ^45^ for the M100 sample. The characteristic peaks were observed at 148 cm^–1^, 198 cm^–1^, 284 cm^–1^, 305 cm^–1^, 405 cm^–1^, 484 cm^–1^, 530 cm^–1^, 704 cm^–1^, and 998 cm^–1^, which corresponds to orthorhombic α-V_2_O_5_ ^46^ for V100. For M100 sample, the high frequency bands at 996 cm^–1^ and 820 cm^–1^ are assigned to asymmetric (*v*_as_ Mo=O) and symmetric (*v*_as_ Mo–O–Mo) stretching.^47^ Frequency bands at 378 cm^–1^ and 337 cm^–1^ are related to the *v*O=Mo=O and δO–Mo–O deformation modes. The frequency band at 292 cm^–1^ is related to δO=Mo=O wagging modes.^47^ The detailed assignment of MoO_3_ is available in the literature,^48,49^ and the Raman bands of MoO_3_ and V_2_O_5_ are summarized in Table 4. For V100 sample, the high-frequency bands at 998 cm^–1^ and 704 cm^–1^ are assigned to asymmetric and symmetric stretching of V_(a)_-O_(3)_-V_(b)_ bridges.^50^ The vibrations of V_(a)_-O_(2a)_ and V_(b)_-O_(2b)_ bonds are found at 530 and 484 cm^–1^; also, ladder-like distortion modes are found at 305 and 284 cm^–1^.^50^ The detailed assignments for V_2_O_5_ are available in the literature.^50–52^ For the intermediate compositions (M80 to M20), the Raman spectra show a significant evolution characterized by peak broadening, reduced intensity, and partial merging of vibrational modes. Unlike the sharp and well-resolved Raman bands observed for M100 and V100, the intermediate films show broadening of the Raman modes. This is due to the increased lattice disorder that arises from the coexistence of MoO_3_ and V_2_O_5_ within a shared oxygen network. The incorporation of Mo and V species with different ionic radii, coordination environments, and bonding preferences leads to the lattice distortion. Frequency bands appearing near 700 and 900 cm^–1^ are assigned to overlapping contributions from Mo=O stretching modes of MoO_3_ and V–O–V or V=O stretching modes of V_2_O_5_. Similarly, the mid-frequency region around 300-500 cm^–1^ shows merged bands associated with O–Mo–O bending, O–V–O bending, and mixed metal–oxygen-metal vibrations. The observed peak broadening and merging of Raman bands in the intermediate compositions are attributed to lattice distortion and mixed Mo–O–V bonding environments, consistent with earlier reports on mixed TMOs.^53,54^

**Figure 5:**
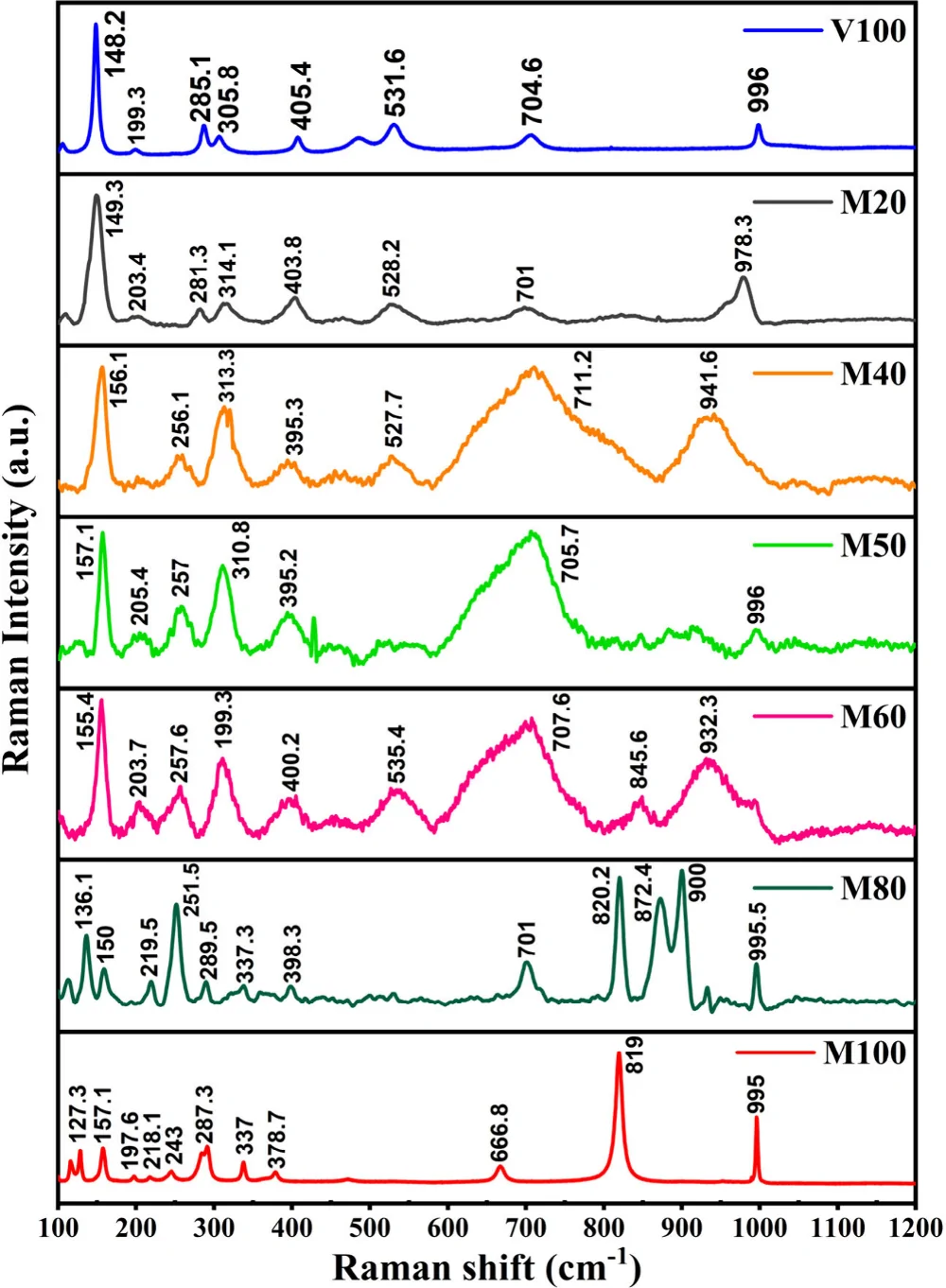
Raman spectra of MoO_3_–V_2_O_5_ thin films across different compositions.

**Table 4:** Summary of Raman Bands Observed in α-MoO_3_ (M100) and V_2_O_5_ (V100)

α-MoO_3_ ^45^	α-MoO_3_ (this work)	α-V_2_O_5_ ^46^	α-V_2_O_5_ (this work)
996	996	992	998
823	820		
667	667	698	704
473	471	524	530
380	378	476	484
376		403	405
366			
334	337	299	305
		281	284
293	292		
285	285	194	198
		142	148
247	243		
216	218	98	-
197	197		
159	158		
129	129		
116	117		
100	-		
89	-		

### 3.4 Morphological Analysis

Figure 6 shows the FESEM morphology of MoO_3_–V_2_O_5_ thin films across all the compositions. V100 sample which corresponds to pure V_2_O_5_ exhibits densely packed microstructure with grain size of about 25 nm. Similarly, M100, which corresponds to pure MoO_3_, shows a compact surface morphology with a larger grain size of about 50 nm presenting higher crystallite size, which indicates efficient grain growth but reduced surface area. In contrast, the intermediate compositions (M20-M80) show a significant morphological variation with the emergence of porous, interconnected nanorod architectures. As MoO_3_ content increases to M40 and M60, the samples exhibit rod like nanostructures that form highly open with enhanced surface area. Figure 6 (i) shows the EDS spectrum of M20 composition. It was used for elemental analysis, and it confirmed the compositional accuracy as shown in Table 1. Elemental mapping analysis Figure 6(j-l) confirms homogeneous and uniform Mo and V elemental distribution throughout the film thickness for all samples.

**Figure 6:**
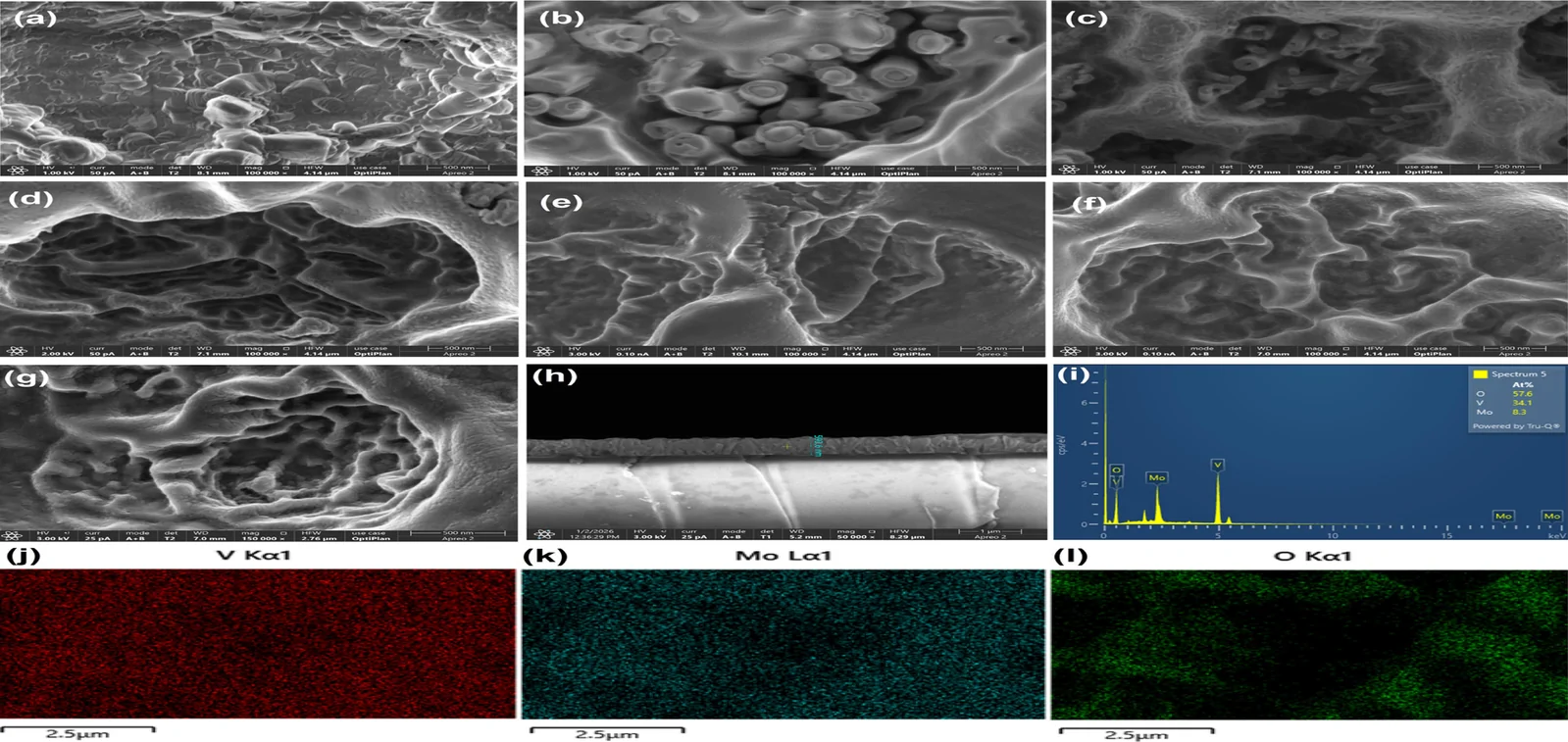
FE-SEM images of MoO_3_–V_2_O_5_ thin films across various compositions: (a) M100, (b) M80, (c) M60, (d) M50, (e) M40, (f) M20, (g) V100, (h) cross-sectional image of M20 composition, (i) EDS spectrum for M20 composition, and (j-l) Elemental mapping of M20 composition.

### 3.5 Electrical Properties

The electrical properties of MoO_3_–V_2_O_5_ thin films were measured using a linear four-probe configuration. All measurements were performed at room temperature under ambient conditions. The sheet resistance R_s_ was calculated using the standard geometric correction factor for an equally spaced linear four probe configuration shown in relation **(7)**. This correction factor accounts for the probe geometry and enables accurate determination of surface electrical properties independent of sample size.^55^ The bulk resistivity was obtained from the relation **(8)**, where *t* is the film thickness.^56^ This procedure yielded resistances ranging from the GΩ for MoO_3_ rich samples to MΩ range for V_2_O_5_ rich samples, consistent with the transition from insulating MoO_3_ to comparatively more conductive V_2_O_5_ rich samples.

7 $$R_{s}=\frac{\pi }{\ln\left(2\right)}R=4.53R$$

8 $$\rho =R_{s}\cdot t$$

The gradual drop in sheet resistance from M100 to V100 as shown in Table 5. MoO_3_-rich samples conduct poorly, and the charge carriers move slowly through the structure due to limited electron density of states.^57^ As the V_2_O_5_ content increases in the films, mixed-valence V^5+^/V^4+^ sites progressively appear within the V_2_O_5_ phase, which substantially improves electron mobility across the lattice. The superior conductivity of V_2_O_5_-rich compositions directly arises from the abundance of redox-active V^5+^/V^4+^ sites that facilitate both electronic conduction and pseudocapacitive charge storage.^58^ The progressive increase in conductivity from M100 to V100 corresponds to an increasing V_2_O_5_ content, demonstrating that V_2_O_5_ oxidation states and their availability dominate the electrical response of the compositional series.

**Table 5:** Measurement of Electrical Parameters from Four-Probe Configuration

Sample	R (Ω)	R_s_ (Ω/□)	ρ(Ω·cm)
V100	1.00 × 10^6^	4.53 × 10^6^	2.69
M20	3.40 × 10^6^	1.54 × 10^7^	9.14
M40	9.50 × 10^6^	4.30 × 10^7^	25.5
M50	1.60 × 10^7^	7.25 × 10^7^	43.03
M60	1.192 × 10^7^	5.40 × 10^8^	320.5
M80	7.90 × 10^8^	3.58 × 10^9^	2125
M100	1.75 × 10^9^	7.93 × 10^9^	4707.2

### 3.6 Electrochemical Analysis

Initial electrochemical characterization of all the prepared thin film electrodes was carried out in aqueous electrolytes (1M KOH and 1M Na_2_SO_4_) to assess their intrinsic electrochemical stability. However, both the electrolytes were found to be unstable with MoO_3_–V_2_O_5_ thin films. In alkaline 1M KOH, rapid degradation of film was observed due to hydroxyl ions, where OH^–^ attacks oxide heterostructure, which leads to the formation soluble molybdate (MoO_4_^–2^) and vanadate (VO_3_^–^) ions shown in **(9)** and **(10)**.

9 $$V_{2}O_{5}\left(s\right)+2{\mathrm{OH}}^{-}\left(\mathrm{aq}\right)\to 2{{\mathrm{VO}}_{3}}^{-}\left(\mathrm{aq}\right)+H_{2}O\left(l\right)$$

10 $${\mathrm{MoO}}_{3}\left(s\right)+2{\mathrm{OH}}^{-}\left(\mathrm{aq}\right)\to {{\mathrm{MoO}}_{4}}^{2-}\left(\mathrm{aq}\right)+H_{2}O\left(l\right)$$

In neutral 1M Na_2_SO_4_ electrolyte, although the hydroxide concentration is negligible, film instability was still observed. This behavior is due to the water reactivity that disrupts the V–O–V and Mo–O–Mo metal oxide frameworks, which leads to the gradual structural degradation of the active material. These limitations made us take a transition to an aprotic organic electrolyte system. The 1 M LiBF_4_ in propylene carbonate was selected as electrolyte, which eliminates water- and hydroxide-driven degradation and also provides an expanded electrochemical stability window. Figure 7 illustrates the schematic representation of symmetric two-electrode configurations. This electrolyte selection also provides an important distinction from many previously reported MoO_3_- and V_2_O_5_-based electrodes evaluated in aqueous electrolytes.^59–62^ The present study instead emphasizes the systematic influence of MoO_3_–V_2_O_5_ composition on the structural and electrochemical behavior of binder-free thin-film electrodes under a consistent non-aqueous electrolyte environment. Consequently, electrochemical characterization for all the samples was done using cyclic voltammetry (CV), electrostatic impedance spectroscopy (EIS), and galvanostatic charge–discharge (GCD) in 1 M LiBF_4_/propylene carbonate using a symmetric two-electrode configuration, ensuring both thermodynamic stability of the thin films and reliable evaluation of their intrinsic charge storage behavior.

**Figure 7:**
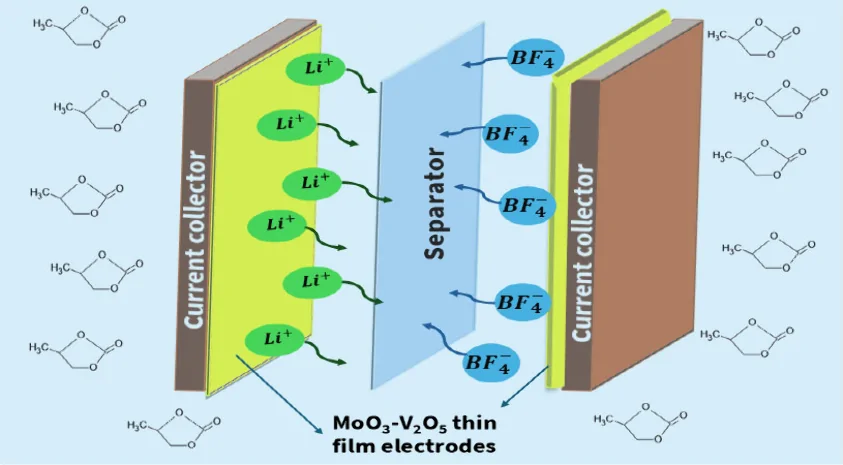
Schematic illustration of symmetric supercapacitor configuration.

#### 3.6.1 Cyclic Voltammetry (CV)

The CV curves of all the samples, recorded in the potential window of 0 to 1.5 V at a scan rate of 100 mV s^–1^, exhibit nearly quasi rectangular and symmetric profiles, which is characteristic of pseudocapacitive charge storage behavior. Such shapes indicate a rapid and reversible redox process with minimal diffusion limitations, suggesting that the thin films provide accessible electroactive sites and efficient ion transport within the probed voltage range.^63^ Figure 8(a) illustrates the CV curves of all the MoO_3_–V_2_O_5_ thin films across different compositions. Among all compositions, it was observed that M20 sample shows the largest integrated area under the CV curve, followed by M40 and V100, which reflects its superior ability to store and deliver charge during cycling. This evolution of the CV response with composition is consistent with previous reports on mixed transition metal oxide systems, where optimized combinations of two oxides often lead to improved electrochemical activity owing to synergistic effects.^63^ The areal capacitance and specific capacitance were calculated using relations **(11)** and **(12)**, where *ΔV* is the potential window, *m* is the mass of the active electrode material (g), *v* is the scan rate, and ∫ *IdV* represents the area under the CV curve.^64^ The active mass loading of each MoO_3_–V_2_O_5_ thin film electrode used in the symmetric two-electrode device configuration was approximately 0.35 mg. As shown in Figure 8(b), the calculated specific and areal capacitances vary systematically across the composition series, with the M20 sample exhibiting the highest capacitance which delivers an areal capacitance of 3.57 mF cm^–2^ and specific capacitance of 10.22 F g^–1^ at a scan rate of 100 mV s^–1^, far exceeding all other compositions. The coexistence of these two oxides MoO_3_ and V_2_O_5_ in M20 sample results in improved ion accessibility and faster charge transfer kinetics, leading to enhanced electrochemical response. In contrast it was observed that the compositions with higher Mo or V content show reduced current response. In pure V_2_O_5_ (V100 sample) strong redox activity was observed with an areal capacitance of 1.58 mF cm^–2^ and specific capacitance of 4.518 F g^–1^ at a scan rate of 100 mV s^–1^, but it performs lower than M20 sample. This confirms that the composition tuning plays a critical role in determining electrochemical behavior.

11 $$C_{areal}^{cv}=\frac{\int IdV}{v\cdot A\cdot \Delta V}$$

12 $$C_{specific}^{cv}=\frac{\int IdV}{v\cdot m\cdot \Delta V}$$

**Figure 8:**
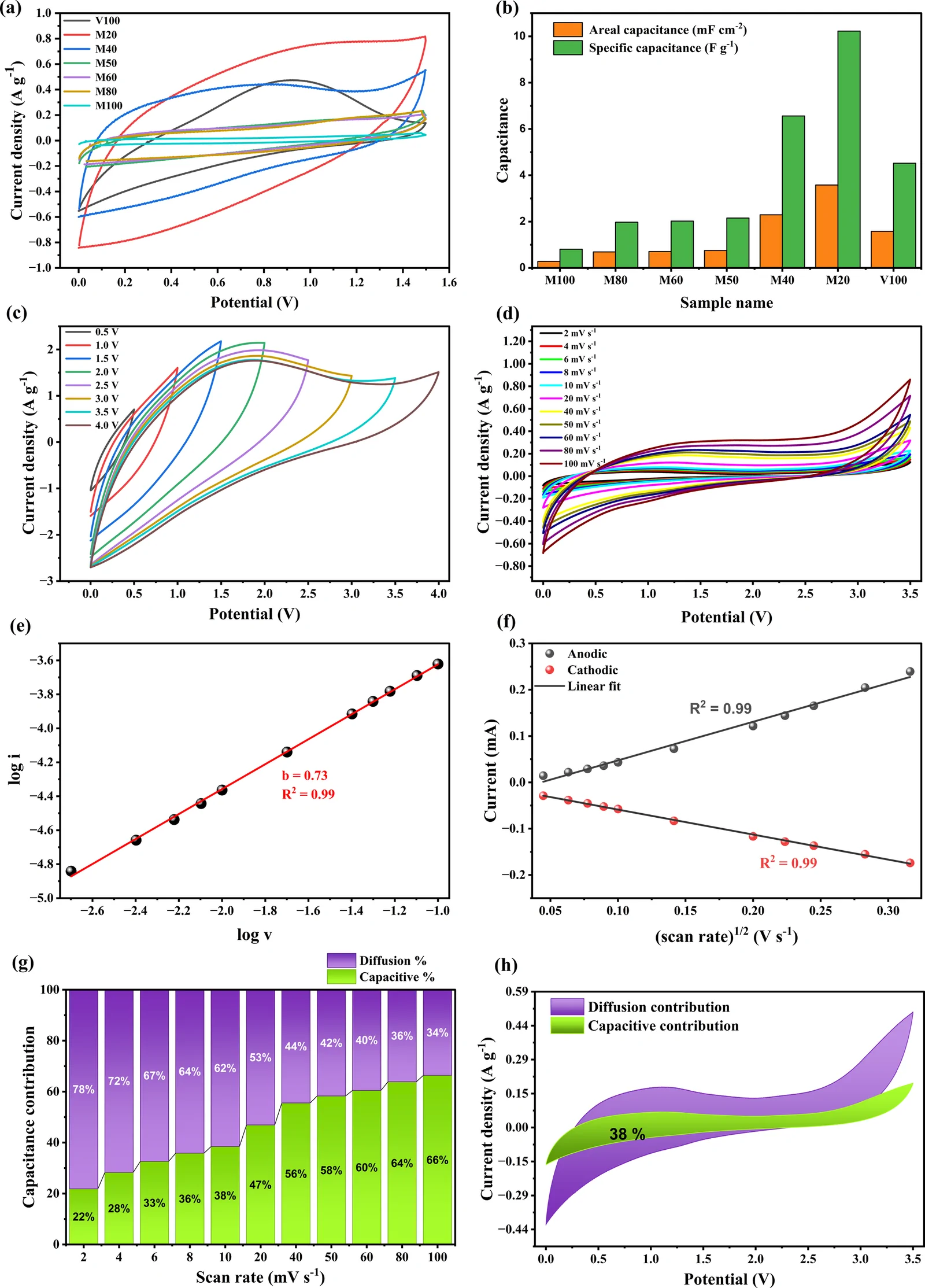
(a) CV curves of all the thin film electrodes across different compositions at 100 mV s^–1^, (b) Calculated specific and areal capacitances for all samples, (c) CV curves of M20 sample at different potential window, (d) CV curves of M20 sample at different scan rates, (e) Logarithmic relationship between peak current (log (i)) and scan rate (log ν), (f) Separation of anodic and cathodic currents plotted against the square root of scan rate, (g) Percentage contribution of capacitive and diffusion controlled processes as a function of scan rate, and (h) Quantitative differentiation of current response into capacitive and diffusion-controlled contributions at 10 mV s^–1^ using Dunn’s method.

Further, the CV curves of the M20 sample recorded at different potential window are shown in Figure 8(c). This dynamic response reflects the reversible redox transitions of molybdenum and vanadium ions, facilitated by intercalation and deintercalation of Li^+^ ions within the MoO_3_–V_2_O_5_ lattice.^65,66^ This ability to operate up to 4 V is particularly significant as it shows the advantage of use of an aprotic organic electrolyte. Unlike aqueous electrolytes which are limited by narrow voltage windows and active material degradation, the organic system enables high voltage operation without hydrolytic instability. Use of an organic electrolyte enables us to use a wide electrochemical potential window up to 4 V, but for reliable and stable operation of all the samples, the electrochemical measurements were confined to a voltage window of 1.5 V. Cyclic voltammetry measurements of the M20 electrodes were further performed over an extended potential window of 3.5 V for different scan rates (2-100 mV s^–1^) as shown in Figure 8(d) to evaluate the accessible charge storage capacity under wide voltage operation. It shows a systematic increase in the current density with increasing scan rate, and at a scan rate of 2 mV s^–1^, it showed a specific capacitance of 46.35 F g^–1^ which can be attributed to the enlarged electrochemical window enabling greater charge accumulation and activation of additional pseudocapacitive redox sites. It showed higher capacitance at a lower scan rate (2 mV s^–1^), which is due to sufficient time for electrolyte ions to diffuse into the electrode and access a larger number of electrochemically active sites.^67^

#### 3.6.2 Charge Storage Mechanism

The charge storage mechanism of the MoO_3_–V_2_O_5_ composite thin film electrodes was analyzed to understand the nature of electrochemical processes and its performance in LiBF_4_/propylene carbonate electrolyte. In general, electrochemical energy storage in transition metal oxide based electrodes arises from a combination of faradaic processes involving reversible redox reactions and non-faradaic processes associated with electric double layer formation at the electrode/electrolyte interface. In this present system both contributions coexist due to the layered structure and multivalent redox activity of MoO_3_ and V_2_O_5_. In MoO_3_–V_2_O_5_ composite the pseudocapacitive behavior is primarily governed by the redox transitions of Mo and V ions represented by **(13)** and **(14)**; also, because of electrolyte insertion and extraction of Li^+^ ions, it happens as shown in **(15)**. Thus, the overall charge storage mechanism involves both surface-controlled reactions and diffusion-limited Li^+^ intercalation within the oxide lattice.^68^

13 $${\mathrm{Mo}}^{6+}+e^{-}↔{\mathrm{Mo}}^{5+}$$

14 $$V^{5+}+e^{-}↔V^{4+}$$

15 $${\mathrm{MoO}}_{3}-V_{2}O_{5}+{\mathrm{x Li}}^{+}+{\mathrm{x e}}^{-}↔{\mathrm{Li}}_{x}\left({\mathrm{MoO}}_{3}-V_{2}O_{5}\right)$$

The b-value is obtained from the power law relation as shown in eq 16, and the log form is given in eq 17

16 $$i=av^{b}$$

17 log(i)=log(a)+blog⁡v

where i is the peak current, v is the scan rate, a is constant, and b is the slope^69^ from the plot shown in Figure 8(e). The b-value obtained was 0.73, which indicates a hybrid charge storage mechanism where both surface controlled capacitive processes and diffusion limited intercalation leads to overall electrochemical response. The relative contributions of capacitive and diffusion controlled processes strongly depend on the scan rate. At lower scan rates, Li^+^ ions have sufficient time to diffuse, which leads to a higher diffusion controlled contribution. At higher scan rates, ion diffusion becomes limited and the charge storage is dominated by surface controlled processes. Based on the combined analyses MoO_3_–V_2_O_5_ thin films exhibit a hybrid charge storage mechanism. The synergetic interaction between MoO_3_ and V_2_O_5_ enhances electronic conductivity and provides multiple redox active sites and facilitates rapid ion transport. The presence of Li^+^ ions in the electrolyte enables reversible intercalation into the layered oxides while the surface area of the thin film promotes capacitive storage.^70^ Dunn’s method was employed to quantitatively distinguish capacitive and diffusion controlled contributions for 10 mV s^–1^ CV curve. In this approach, the measured current (i) at a given potential is separated into two components as shown in eq 18

18 $$i\left(v\right)=k_{1}v+k_{2}v^{1/2}$$

where the term *k*_1_ *v* represents the capacitive-controlled contribution and *k*_2_ *v* ^1/2^ corresponds to the diffusion-controlled contribution.^71,72^ The quantitative separation of these contributions was carried out at different scan rates, and the calculated contributions are illustrated in Figure 8(g).

#### 3.6.3 Electrochemical Impedance Spectroscopy (EIS)

EIS tells us about the charge transfer kinetics and mechanistic data of various electrochemical systems.^73^ Figure 9 shows the EIS measurements of all the prepared MoO_3_–V_2_O_5_ thin films across different compositions. EIS was conducted in the frequency range of 0.1 Hz to 1 kHz. Nyquist plots for all of the samples show the composition-dependent change in the impedance response, which reflects systematic changes in the interfacial resistance and capacitive behavior across the compositional series. The EIS data were fitted using appropriate equivalent circuits, where R_s_ is the series/solution resistance, R_ct_ is the charge-transfer resistance, CPE_dl_ is the constant phase element for the electric double layer, and CPE_diff_ is the constant phase element representing diffusion-controlled ion transport. The presence of CPE_diff_ in the V_2_O_5_-rich compositions (V100-M40) is consistent with the well-known layered structure of V_2_O_5_, whose van der Waals interlayer galleries readily accommodate bulk cation intercalation, giving rise to a diffusion-controlled (Warburg type) low-frequency response. In contrast, the MoO_3_-rich compositions (M50-M100) do not require a diffusion element, suggesting charge storage dominated by surface/near-surface redox (pseudocapacitive) processes rather than bulk ion insertion, adequately described by a single time constant.^74^ The fitting parameters are summarized in Table 6. R_s_ remains relatively constant for most compositions, indicating comparable electrolyte resistance and electrical contact throughout the measurements. In contrast, R_ct_ shows a compositional dependence. The V_2_O_5_-rich compositions (V100-M40) show comparatively lower R_ct_ values, indicating faster interfacial charge transfer kinetics, whereas the MoO_3_-rich compositions (M50-M100) show an increase in R_ct_ values, reflecting sluggish charge transfer and increased interfacial resistance.^75,76^

**Figure 9:**
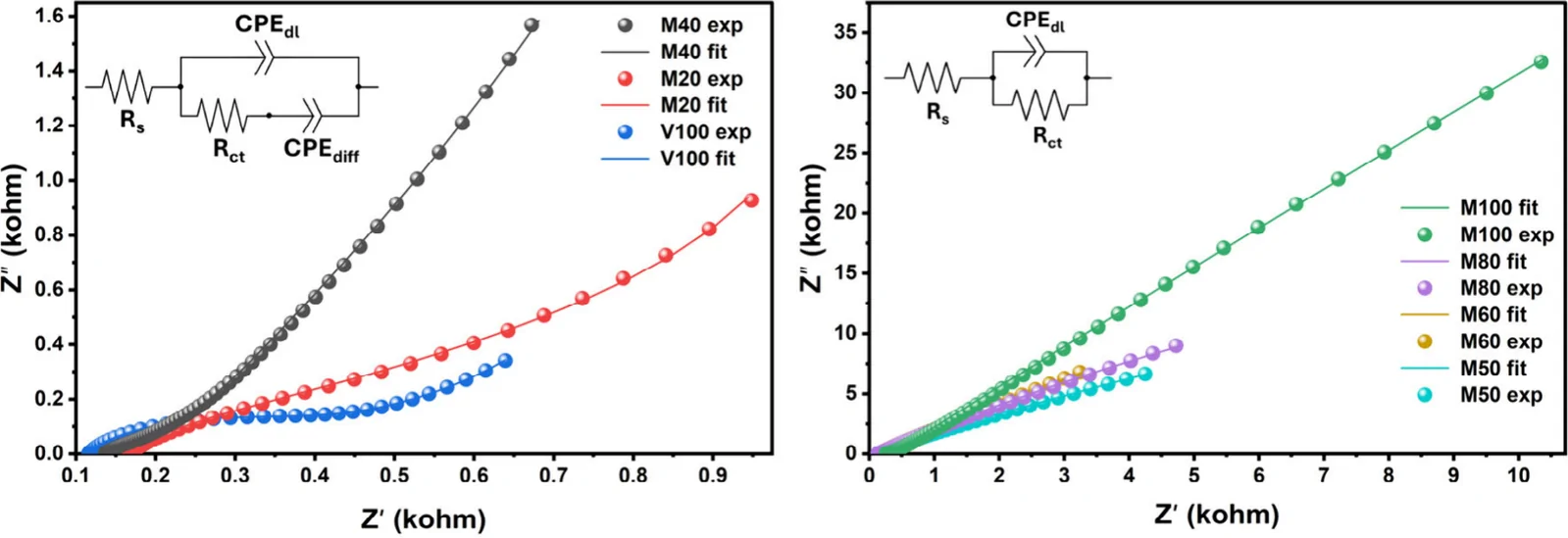
Nyquist plots of the MoO_3_–V_2_O_5_ thin-film electrodes (a) for V100, M20, and M40 and (b) for M50, M60, M80, and M100 samples.

**Table 6:** EIS Fitting Parameters of MoO_3_–V_2_O_5_ Thin-Film Electrodes

			CPE_dl_		CPE_diff_	
Sample	R_s_ (Ω)	R_ct_ (Ω)	P_dl_ × 10^–5^ (F s^n–1^)	n	P_diff_ × 10^–5^ (F s^n–1^)	n
V100	115.31	2.77 × 10^2^	1.32	0.74	1.51	0.76
M20	122.65	2.48 × 10^2^	4.94	0.78	5.14	0.84
M40	134.61	7.62 × 10^2^	9.55	0.47	8.57	0.86
M50	126.62	7.47 × 10^4^	16.7	0.70	-	-
M60	132.51	65.6 × 10^5^	18.2	0.77	-	-
M80	131.57	19.4 × 10^5^	14.1	0.76	-	-
M100	186.16	72.9 × 10^5^	4.17	0.74	-	-

#### 3.6.4 Galvanostatic Charge–Discharge (GCD)

Galvanostatic charge–discharge (GCD) measurements were carried out to evaluate capacitive performance of the MoO_3_–V_2_O_5_ composite thin film electrodes. The GCD curves of all the prepared thin film electrodes were studied at a current density of 1.43 A g^–1^ at an operating voltage of 1.5 V, and the charge–discharge patterns are shown in Figure 10(a). It was observed that M20 thin film electrodes show a longer discharge time compared to other electrodes indicating an optimized composition for enhanced electrochemical performance. MoO_3_–V_2_O_5_ thin films show a nonlinear charge–discharge curve; therefore, specific and areal capacitances were calculated using relations **(19)** and **(20)** where *I* is discharge current, *t*_*d*_ is discharge time (s), *m* is active material mass (g), and (*V*_*f*_ – *V*_*i*_) is the potential window (V).^77,78^

19 $$C_{s}^{\gcd}=\frac{2I\int Vdt}{m{\left(V_{f}-V_{i}\right)}^{2}}$$

20 $$C_{a}^{\gcd}=\frac{2I\int Vdt}{A{\left(V_{f}-V_{i}\right)}^{2}}$$

**Figure 10:**
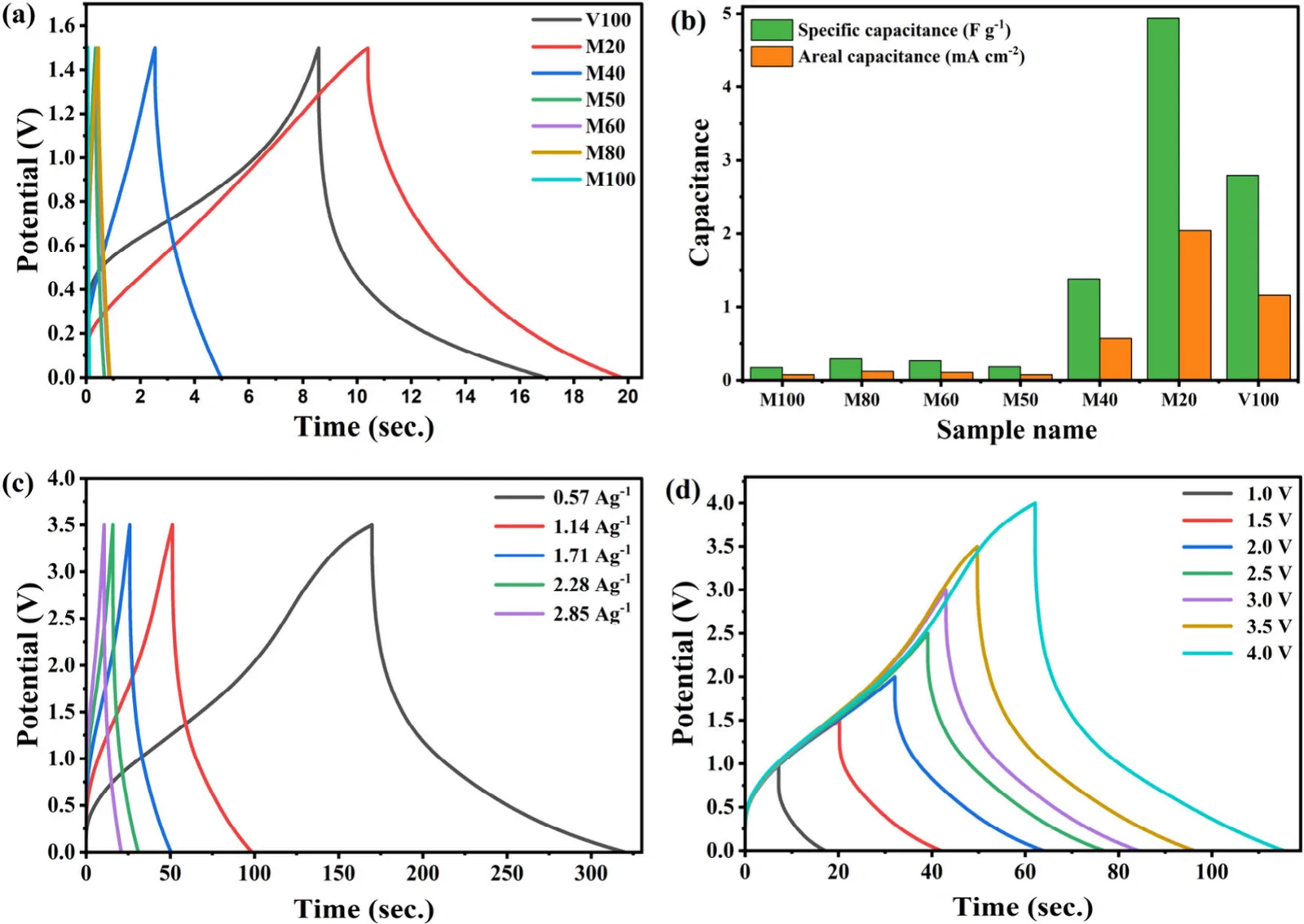
(a) GCD curves of all the thin film electrodes across different compositions at 1.43 A g^–1^, (b) Calculated specific and areal capacitances for all samples from GCD, (c) GCD curves of M20 sample at different current densities; (d) GCD curves of M20 sample at different potential range.

V_2_O_5_ rich sample (M20) exhibited the highest *C*_*s*_^*gcd*^ of 4.94 F g^–1^ reflecting abundant V^5+^/V^4+^ redox sites while pure MoO_3_ (M100) showed minimal *C*_*s*_^*gcd*^ of 0.15 F g^–1^ consistent with its low conductivity. M20 sample overperformed V100, which may be due to the synergetic effects of both MoO_3_ and V_2_O_5_. Further GCD measurements were performed on optimized M20 sample at different current densities and over an extended potential window as shown in Figure 10(c, d). The GCD curves recorded at different current densities demonstrated good rate capability and structural stability. At a current density of 0.57 A g^–1^, the M20 electrode delivers a specific capacitance of 25 F g^–1^, corresponding to an energy density of 42.5 Wh kg^–1^ and a power density of 997 W kg^–1^, reflecting efficient charge storage under low rate operating conditions. Furthermore, the electrode exhibits stable performance over a wide potential window up to 4 V in LiBF_4_/propylene carbonate electrolyte which increases the energy density.

The electrochemical stability of the optimized M20 MoO_3_–V_2_O_5_ thin film electrodes was done using repeated GCD cycling over 10,000 cycles at a current density of 1 A g^–1^. Cycling stability is important as it tells about the structural stability of the electrode material and the reversibility of the charge storage processes. The M20 electrode shows capacitance retention of 89.7% after 10,000 cycles indicating only minimal loss of the initial capacitance over long cycling. In addition the electrode shows a high coulombic efficiency of 98.4% indicating highly reversible charge–discharge processes as shown in Figure 11(a). The GCD curves before cycling and after 10,000 cycles have nearly overlapping shapes with a slight increase in the discharge time after prolonged cycling as shown in Figure 11(b). This increase supports the activation process, indicating improved ion transport pathways within the electrode. A similar trend was observed in CV curves recorded before and after cycling. Figure 11(c) shows an increase in the enclosed area, which tells about the improved electrode-electrolyte interaction and enhanced accessibility of active sites due to structural rearrangement during cycling. The Nyquist plots obtained from EIS measurments before and after cycling (Figure 11(d)) show negligible change in series resistance (R_s_) indicating stable electrolyte conductivity. A slight variation in charge transfer resistance (R_ct_) is observed, which can be associated with interfacial modification and activation of the electrode surface. The preservation of the linear low-frequency region confirms that Li^+^ ion diffusion kinetics remain effective even after extended cycling. Overall the combined results show that M20 MoO_3_–V_2_O_5_ thin film electrode exhibits excellent electrochemical stability.

**Figure 11:**
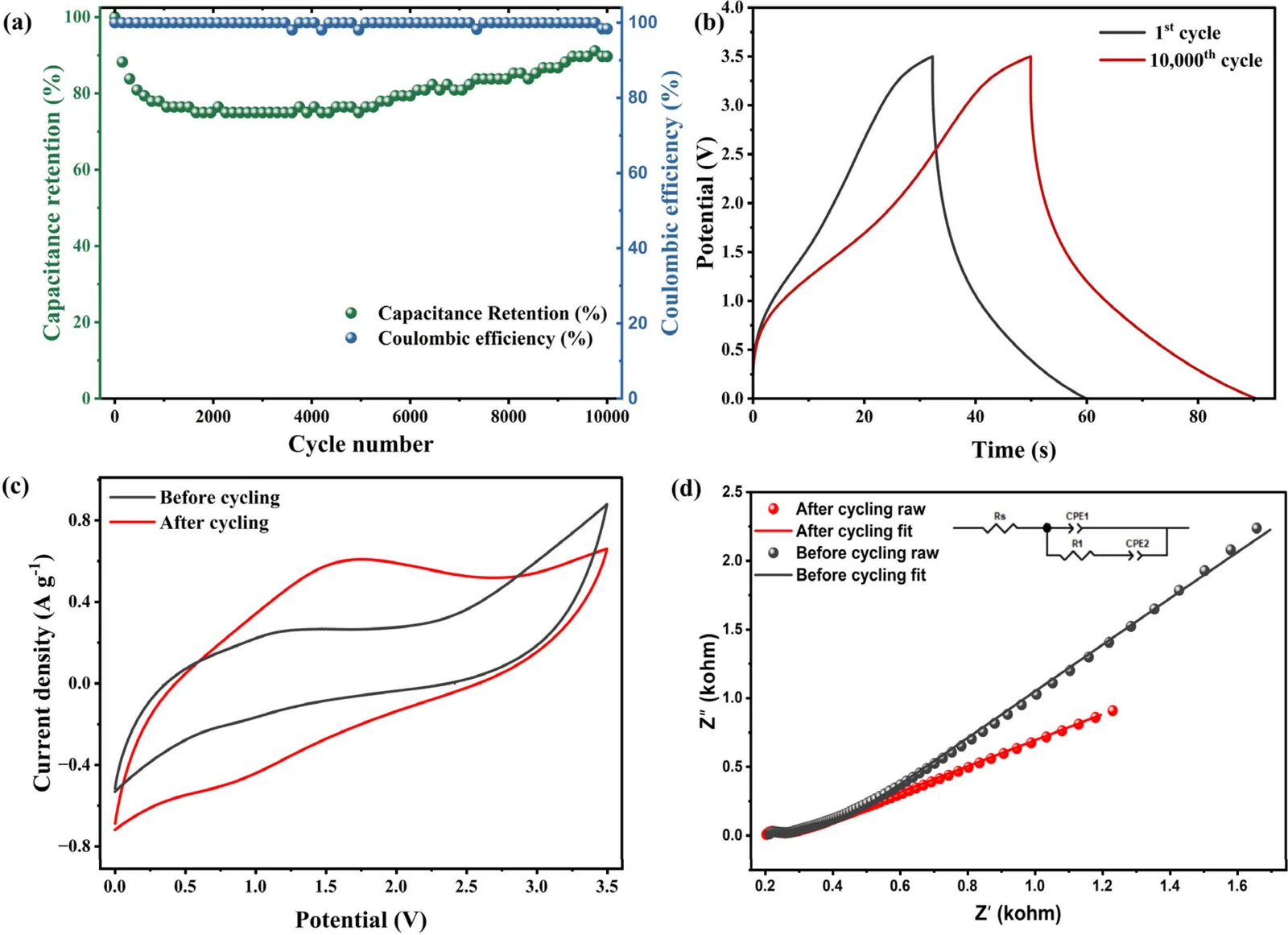
(a) Capacitance retention (%) and coulombic efficiency (%) as a function of cycle number up to 10,000 cycles at 1 A g^–1^, (b) Comparison of GCD profiles before cycling and after 10,000 cycles, (c) CV curves recorded before and after cycling, (d) Nyquist plots (EIS) before and after cycling along with equivalent circuit fitting.

## 4 Conclusion

In summary, MoO_3_–V_2_O_5_ thin films with precisely controlled Mo:V ratios were successfully synthesized using a spray pyrolysis approach, enabling a systematic investigation of composition property relationships. Structural and spectroscopic analyses confirmed the coexistence of MoO_3_ and V_2_O_5_ phases at intermediate compositions, with the formation of Mo–O–V linkages inducing lattice distortion and modifying the local electronic environment. Electrochemical studies revealed that aqueous electrolytes lead to rapid degradation of the films due to hydroxyl-driven reactions, whereas the use of an aprotic organic electrolyte (1 M LiBF_4_ in propylene carbonate) ensured electrochemical stability and enabled symmetric two-electrode supercapacitor operation over an extended potential window. Among the investigated compositions, the film having Mo to V ratio as 20:80 exhibited the highest specific capacitance. The enhanced charge storage relative to pristine V_2_O_5_ is attributed to the synergistic interplay between MoO_3_ and V_2_O_5_ phases, wherein complementary redox activity and improved charge transport collectively contribute to higher capacitance. Electrochemical impedance spectroscopy further indicated reduced charge transfer resistance and more ideal capacitive behavior in V_2_O_5_ rich compositions. Also, it showed excellent long term stability retaining 89.7% of its initial capacitance after 10,000 GCD cycles along with coulombic efficiency of 98.4%. The minimal change in GCD and CV profiles after prolonged cycling confirms the preservation of the electrode’s electrochemical behavior and structural integrity. Future efforts will focus on increasing active mass loading, optimizing film thickness, and extending this compositional strategy to asymmetric device configurations. Overall, this work highlights the critical role of synergistic phase coexistence and appropriate electrolyte selection in enhancing the electrochemical performance of transition-metal oxide-based supercapacitors.

## Supplementary Material



## Notes

EDS spectra and elemental mapping of the electrode samples (PDF)
