# Supplementary material for: Composition-Dependent Pseudocapacitive Behavior of MoO3–V2O5 Composite Thin Films in an Aprotic Electrolyte
Source: ACS Omega. 2026 Sep 16;11(38):56776–89. doi: 10.1021/acsomega.6c02074 (PMC13629382; doi:10.1021/acsomega.6c02074)
Supplement: Supplementary file 1 [file ao6c02074_si_001.pdf]

Composition Dependent Pseudocapacitive Behavior of MoO_3–V_2O_5 Composite Thin Films in an Aprotic Electrolyte

Nimbure Revansiddappa [a], Ramachandra Gokul[a], Boodu Anuragh[a], Muthu Selvakumar [a] and MG Mahesha [a, *]

[a] Manipal Institute of Technology, Manipal Academy of Higher Education, Manipal 576104, India

*Corresponding Author.
Email: mahesha.mg@manipal.edu

Supplementary Information

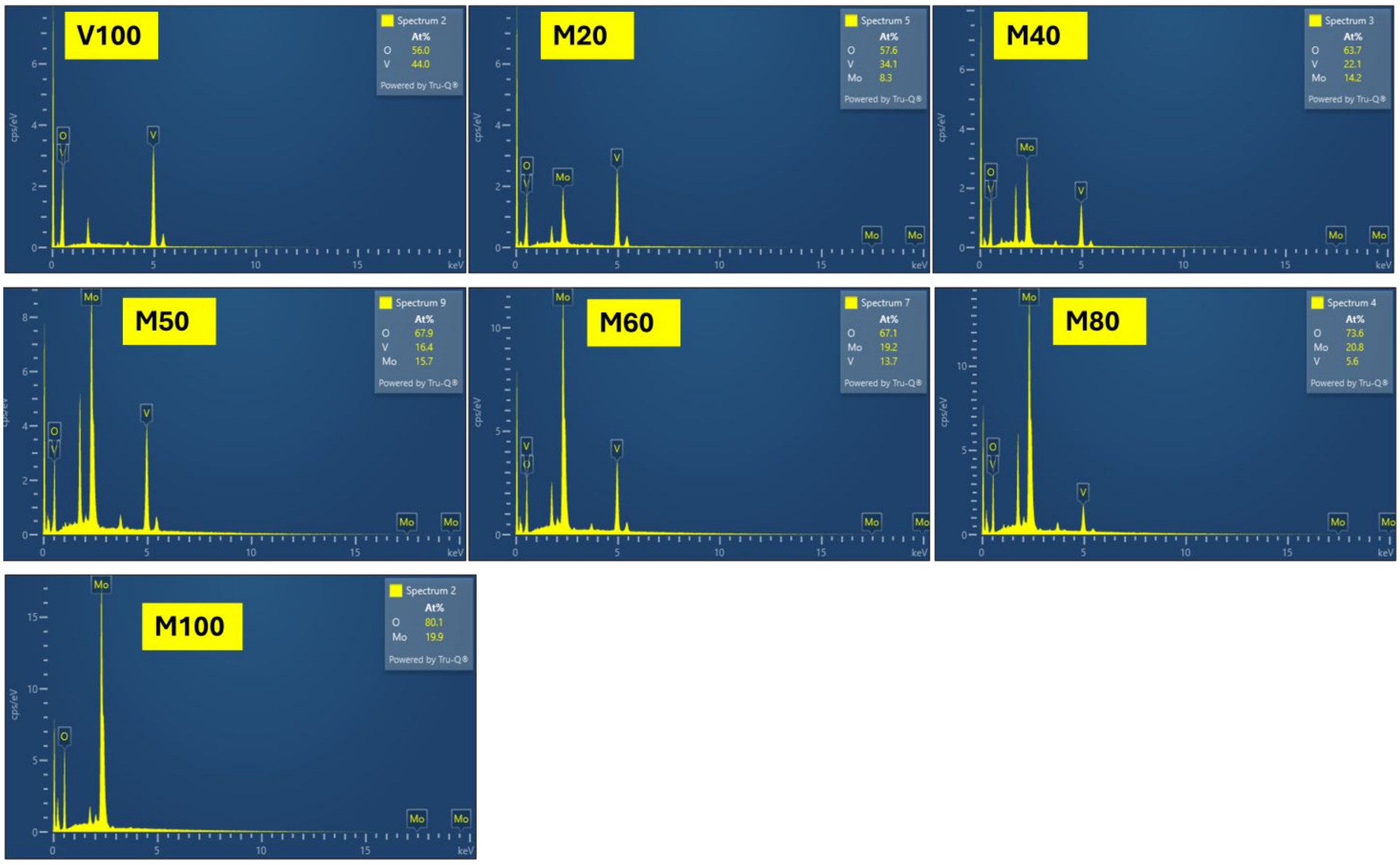

Fig. S1: EDS spectra of electrode samples having different compositional ratios of MoO3 and V_2O_5

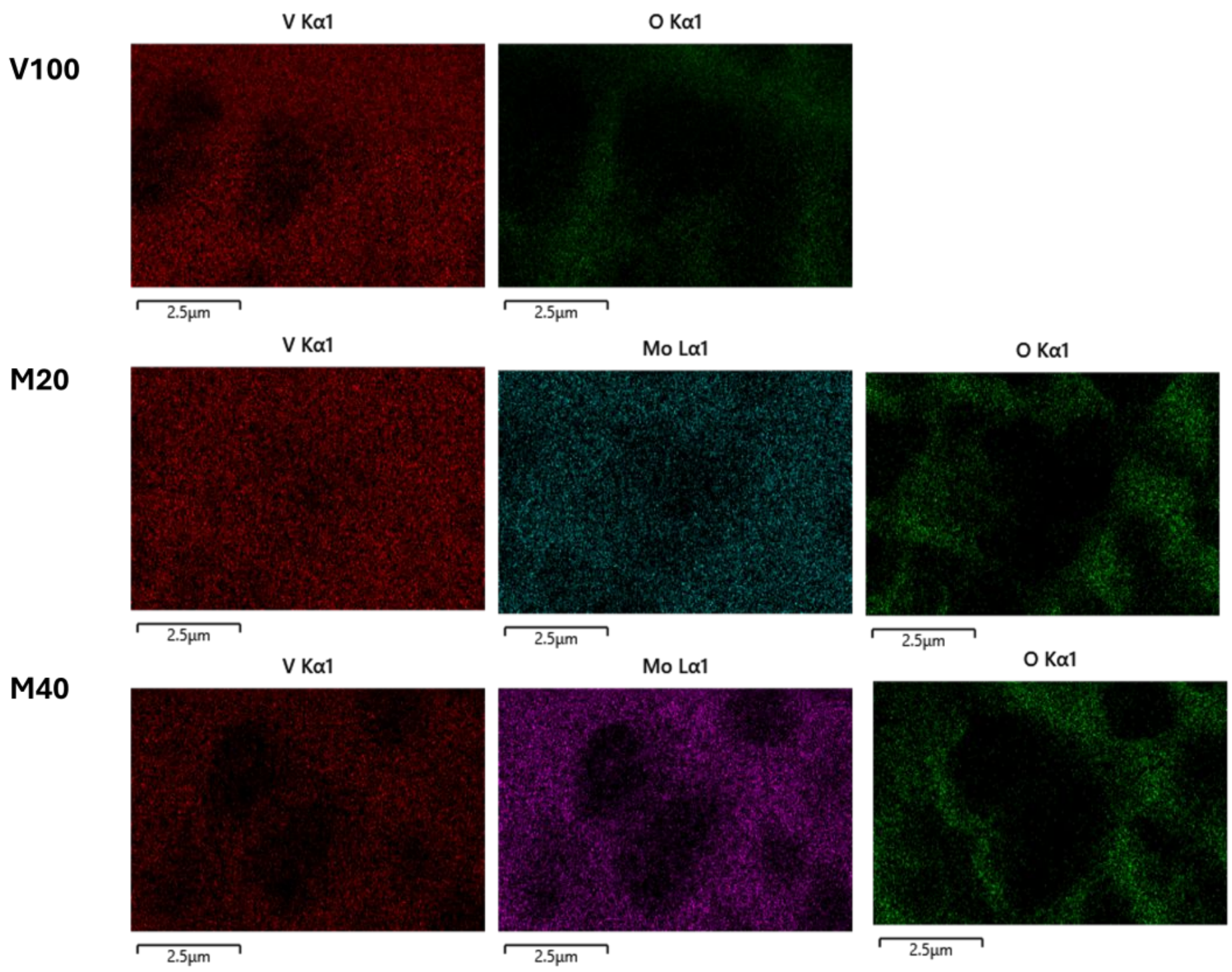

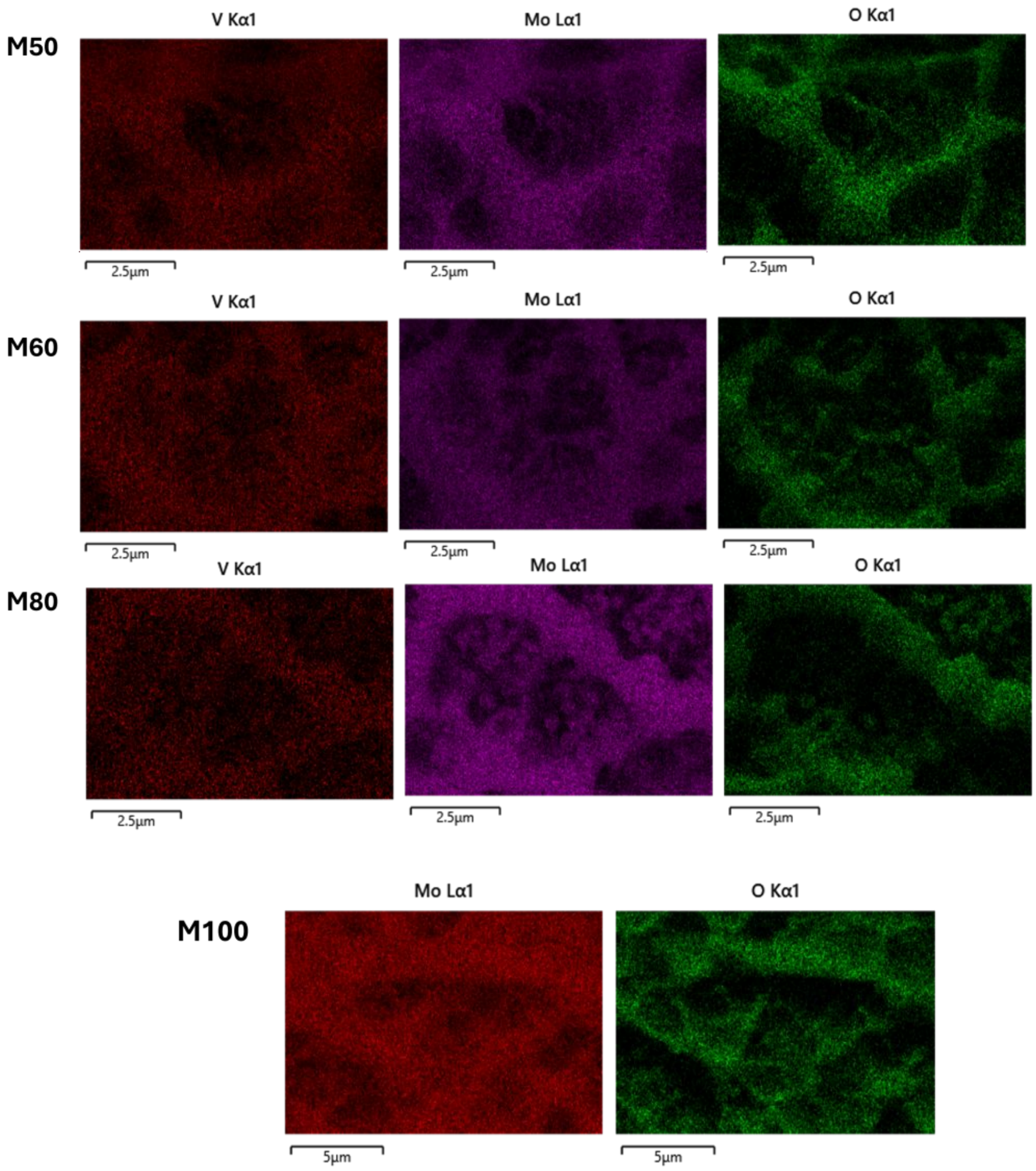

Fig. S2: Elemental mapping of electrode samples having different compositional ratios of MoO3 and V_2O_5 recorded using K_α from V & O, L_α from Mo.
